# The transfer from survey (map-like) to route representations into Virtual Reality Mazes: effect of age and cerebral lesion

**DOI:** 10.1186/1743-0003-8-6

**Published:** 2011-01-31

**Authors:** Laura Carelli, Maria Luisa Rusconi, Chiara Scarabelli, Chiara Stampatori, Flavia Mattioli, Giuseppe Riva

**Affiliations:** 1 Department of Human Science, University of Bergamo, Bergamo, Italy; 2 Applied Technology for Neuropsychology - Laboratory, Istituto Auxologico Italiano, Milano, Italy; 3 Neuropsychological Rehabilitation, Spedali Civili, Brescia, Italy

## Abstract

**Background:**

To go from one place to another, we routinely generate internal representations of surrounding spaces, which can include egocentric (body-centred) and allocentric (world-centred) coordinates, combined into route and survey representations.

Recent studies have shown how both egocentric and allocentric representations exist in parallel and are joined to support behaviour according to the task.

Our study investigated the transfer from survey (map-like) to route representations in healthy and brain-damaged subjects. The aim was two-fold: first, to understand how this ability could change with age in a sample of healthy participants, aged from 40 to 71 years old; second, to investigate how it is affected after a brain lesion in a 8 patients' sample, with reference to specific neuropsychological frames.

**Methods:**

Participants were first required to perform the paper and pencil version of eight mazes, then to translate the map-like paths into egocentric routes, in order to find the right way into equivalent Virtual Reality (VR) mazes.

Patients also underwent a comprehensive neuropsychological evaluation, including a specific investigation of some topographical orientation components.

**Results:**

As regards the healthy sample, we found age-related deterioration in VR task performance. While education level and gender were not found to be related to performance, global cognitive level (Mini Mental State Examination), previous experience with computer and fluidity of navigation into the VR appeared to influence VR task results.

Considering the clinical sample, there was a difficulty in performing the VR Maze task; executive functions and visuo-spatial abilities deficits appeared to be more relevant for predicting patients' results.

**Conclusions:**

Our study suggests the importance of developing tools aimed at investigating the survey to route transfer ability in both healthy elderly and clinical samples, since this skill seems high cognitive demanding and sensitive to cognitive decline.

Human-computer interaction issues should be considered in employing new technologies, such as VR environments, with elderly subjects and neurological patients.

## Background

The ability to find the right way into a novel or familiar environment is a complex and multi-componential function [1,2].

To go from one place to another, we routinely generate internal representations of surrounding spaces, which can include egocentric (body-centred) and allocentric (world-centred) coordinates [3,4]

Allocentric (world-centred) coordinates refer to salient landmarks and their relative positions, whereas egocentric (body-centred) coordinates rely on sensorimotor indexes of head, trunk and body motion, updating the oncoming information according to subjects' movements into the environment. Cognitive maps resulting from these two different ways of selecting and combining spatial topographical information are, respectively, named "survey" and "route" representations. Whereas survey representation allows preserving Euclidean relationship between places in the environment, route representations specify fixed sequences of salient landmarks, describing a starting point, a goal and a direction of movement [5].

In clinical conditions, several topographical impairments, involving, for example, a loss of sense of familiarity for known environments, landmark recognition deficits or problems in memory for complex scenes, can be observed.

There is a great amount of literature describing topographical disorientation after cerebral lesions [6-8]. A loss of ability in wayfinding and place learning has been observed after diffuse impairment of the cerebral cortex (as in neurodegenerative disorders), or after focal lesions, in particular affecting the right cerebral cortex. In the first case, general impairments in attention, memory and perceptual abilities lead to the inability to find the right way into known and unknown places. Specific circuits and structures play an important role in topographical orientation, in particular medial temporal lobe regions, including hippocampus, parahippocampus, lingual gyrus and retro-splenial cortex [9-15].

Several studies have reported that also not pathological aging can impair topographical abilities [16-20].

Age-related differences in cognitive functioning refer to the ability to pay attention and infer information from the world, learn and memorize, solve problems and make decisions. Although the various components of memory do not suffer a homogeneous decline, normal age-related cognitive decline mainly affects information processing speed, attention, ability to inhibit irrelevant or distracting information and working memory capacity. As a consequence, age differences emerge when demanding storage and processing of information are simultaneously required [21].

Paper and pencil tests and ecological ad hoc experimental situations are commonly used in order to evaluate topographical orientation abilities. In particular, ecological methods provide interesting information about strategies used by patients to explore a natural environment. On the other hand, the observation of a patient in daily contexts generates many issues about the reliability and interpretation of collected data. In order to avoid biases, the design of an effective assessment tool, that links a reliable evaluation methodology with a more situated observation of spatial behaviours, is needed.

An interesting tool to investigate topographical difficulties, by integrating both reliability and ecological validity requirements, is represented by Virtual Reality (VR) technology. VR offers both realness and experimental control, providing a high dynamic and interactive representation of a complex environment [22-27].

Recent studies have started to employ VR technology for recreating real or possible situations, in order to investigate wayfinding, place learning and topographical memory in both healthy elderly and brain-damaged subjects. For example, a VR human equivalent of the Morris water maze task has been extensively employed [28]. In this computer-generated virtual space subjects are required to search for a platform hidden beneath the surface of a circular pool. Since the platform cannot be seen directly, the subject must locate the platform position with reference to external cues throughout the environment and remember its location across a series of trials.

Moffat and Resnick (2007) utilized the Virtual water maze to investigate place learning in elderly adults [29]. They found that a good performance in spatial navigation was associated with high executive skills (planning, strategy selection and working memory abilities), spatial memory and speed of processing, all dependent from pre-frontal circuits and proved to be age-sensitive abilities.

Skelton et al (2000) used an equivalent virtual environment (VE) to evaluate place learning abilities in traumatic brain injury subjects [30]. They found that patients were impaired in the VR task, as well as they were during way-finding real life situations, due to an inability to form, remember and use cognitive maps, in consequence to a frontal lesions.

Other studies, aimed at highlighting dissociations between egocentric and allocentric spatial processes, have developed VEs in order to separately investigate these two aspects [31].

However, recent studies have shown that both egocentric and allocentric representations exist in parallel and are joined to support behaviour according to the task [4]. An example of this relation is represented by the typical situation of a tourist with a map of a town he/she's visiting for the first time, where the comparison between the schematic representation of the environment into viewer-dependent coordinates and the reversal process, that is the transfer of the actual egocentric coordinates on the map of the city, are constantly performed. The transfer from survey-like information into route (egocentric) coordinates has not been yet adequately studied, especially if one considers the role played by this ability in everyday life activities requiring topographical orientation skills.

Our study aims to investigate the transfer from survey to route representations into a virtual environment, by highlighting how physiological aging and brain damage affect this mental process.

## Methods

### Participants

#### Healthy controls

40 subjects (15 males, 25 females) aged from 40 to 71 years (mean age: 53.65) without a history of neurological or psychiatric disorder were studied.

The MMSE (Mini Mental State Examination) was administered, and subjects who scored less than 24 (cut off score) were excluded.

After a complete description of the study to the subjects, informed written consent was obtained.

#### Patients

The patient group was composed of 8 patients (5 males, 3 females), aged from 42 to 71 years (mean age: 60.62), with focal brain lesions (3 left sided, 3 right sided, 2 with bilateral lesions) due to vascular accident (see Table 1).

**Table 1:** P&P and VR results of Healthy subjects, divided into age groups

HEALTHY SUBJECTS (AGE GROUPS)		P&P_TOT*	P&P T_TOT**	VR_TOT***	VRT_TOT***
	Mean	8	7,23	4,73	53,2
	
**40-49**	N	15	15	15	15
	
	SD	0	3,68	2,18	15,34

	Mean	8	14	3,50	66
	
**50-59**	N	14	13	14	14
	
	SD	0	7,67	2,59	5

	Mean	8	11,75	1,90	63,5
	
**60-71**	N	11	11	11	10
	
	SD	0	7,38	2,25	16,7

	Mean	8	10,78	3,52	60,48
	
**TOTAL**	N	40	39	40	39
	
	SD	0	6,84	2,56	14,04

*Total number of P&P mazes completed

**Total P&P execution times

***Total number of VR mazes completed

****Total VR execution times

Subjects older than 71 years or with a history of psychiatric or other neurological disease were excluded. Subjects were assessed at least 12 months after onset of clinical symptoms.

### Neuropsychological Assessment

A neuropsychological battery was built according to theoretical and empirical considerations. In particular, we selected standardized measures assessing general cognitive skills, together with tests that have been empirically demonstrated, in previous studies, to be related to spatial navigation and for which normative data have been collected [8].

For general cognitive level, Mini Mental State Examination [32] and Progressive Raven's Matrices (PM 47) [33] were employed. Token test [34], phonological and semantic Fluencies [35] were submitted for verbal abilities evaluation. Verbal memory was assessed through Digit Span (short-term memory) [36] and Short Story Recall (long-term memory) [37], whereas visuo-spatial memory was evaluated by means of Corsi's span [36] (short term memory), Corsi Supra Span [34] and Rey's Copy Recall [37] (long term memory). Attention and executive functions were evaluated through the Trial Making test (divided attention) [38], attentive matrices (selective attention) [34] and the Tower of London test (planning abilities) [39]. Spatial ability evaluation was composed by the Rey's complex figure copy [37], Benton's line orientation test, for line orientation judgement evaluation [40] and the Elithorn's Perceptual Maze test [34], for visuo-spatial planning evaluation. Besides, Manikin's test [41] was administered in order to evaluate right-left orientation ability, whereas geographical knowledge was measured by means of Italy Map test [34]. Finally, the presence of unilateral spatial neglect was assessed by means of the Behavioural Inattention test (BIT) [42].

### The VR-Maze Test

**Figure 1:**
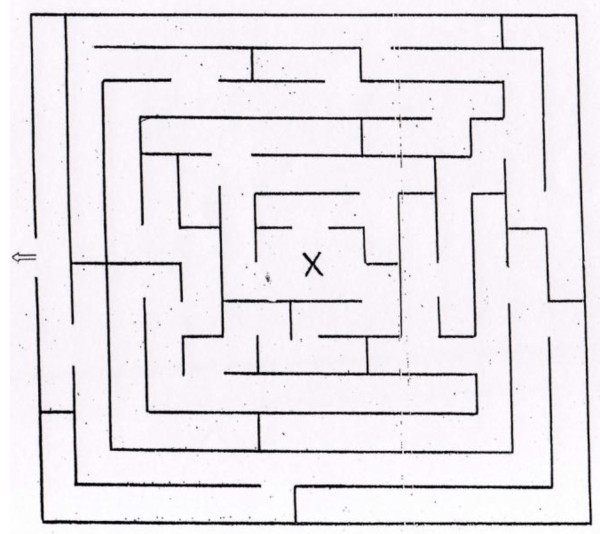
**An example of WISC - R P&P mazes employed in the study**.

**Figure 2:**
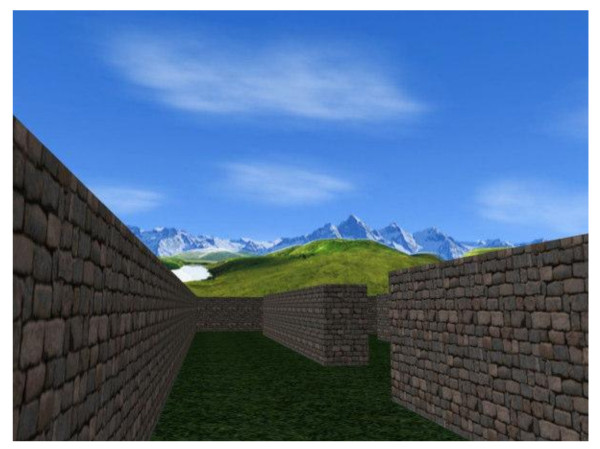
**A screenshot of the third VR Maze**.

The VR-Maze test is based on the Wisc-R Maze subtest (see Figure 1) [43]. It is composed by 8 different virtual mazes (see Figure 2), which are the equivalent VR version of the Wisc-R paper and pencil (P&P) mazes.

First, patients are asked to perform the paper and pencil version of eight mazes, and after to use them in order to find the right way into the equivalent egocentric VR maze version. In order to accomplish this task, each segment of P&P survey paths must be translated into equivalent right and left turns in order to navigate into the VR mazes and find the exit point.

All Mazes lack any kind of landmark, except for a sun shining in the sky at a fixed point. Mountains surround all over the mazes' walls. Subjects may navigate into the VR mazes by means of the keyboard; left-right, up-down and forward-backward movements are allowed and a standard speed of walk is maintained. In order to find the exit point, a maximum time is allowed, then the trial is automatically terminated and subjects enter the following maze.

Mazes (both P&P and VR) differ with respect to global difficulty level. In particular, the third maze is more complex, with a high number of left and right turns whereas the sixth maze requires a 180° turn around oneself, at the starting point, in order to take the correct direction toward the exit point, with a greater cognitive load required not to get lost at the first steps.

## Procedure

Healthy subjects recruited for the study were informed about the experimental procedure and the MMSE was administered. Subjects who scored above the cut off at MMSE evaluation (cut off: 24) were included.

Participants were then asked about their previous experience with computers, answering "Yes" if they were accustomed to daily using laptop computer for professional purposes and/or for play and "No" if they were not ("Experience" parameter).

Prior to Virtual Maze testing, a pre-training phase was provided in order to familiarize with the VE and with the use of keyboard for movement. This was accomplished with an initial period of experimenter instruction, followed by a period of free exploration of a VE using the keyboard. When the participants were comfortable with the keyboard and had satisfactorily demonstrated their ability to guide themselves to targets designated by the experimenter, they were entered into the VR task. Participants were required to reach this competency in less than 10 minutes in order to enter into the experimental phase.

After that, participants were asked to complete each of the eight P&P mazes, by tracing the correct path from the starting point to the exit; a sun was drawn on the top of the paper, showing the north position. During each maze execution, the experimenter recorded execution times and task correctness. After having completed the P&P version, subjects were ready to access into the VR version of mazes.

During the VR task execution, subjects could look at each paper, providing survey-like information (independent from the subject position, being depicted from on-high perspective) to be translated into right and left turns during VR navigation. Besides, they were invited, by the tester, to look at the sun, displayed in the sky during the navigation, as a fixed point useful to orient if they got lost.

Task variables (execution times and correctness of execution, as indicated by the score 1, when maze exit was detected into the maximum time, or score 0, in the opposite case) were recorded for each of the eight mazes; the experimenter also noted qualitative aspects (such as strategies employed to solve the task, perseverative errors and random turns) and verbal comments. Besides, the fluidity of navigation into the VR environment was evaluated with a three level scale: 1 was assigned if participants used all training phase times in order to learn basic movements into the VR, showing slow and not fluid navigation during the task execution; 2, when participants used only one finger at time and 3, when they used more fingers at time to move forward and backward into the VR.

MMSE, P&P and VR maze tasks were administered in a unique session, which took about one hour and a half. For statistical analysis, participants were divided, according to their MMSE score, in two groups (full score: 30 points; below full score group: less than 30 points).

The same procedure was maintained with patients' group, who also performed an additional neuropsychological assessment before starting the VR task. Two sessions were scheduled for each patient: in the first session the informed consent was obtained and the neuropsychological battery was administered; in the next session, P&P and VR mazes were submitted. Exclusion criteria were similar to that described for control subjects as regards the ability of navigation into the VE. The overall experimental procedure took about three hours for each patient.

### Statistical analysis

There were 3 overarching aims of the study: 1) to investigate basic age differences in Virtual Maze performance; 2) to investigate how cognitive impairment, due to cerebral lesions, can influence Virtual Maze performance; and 3) to compare healthy subjects and patient performances.

Healthy subjects' and patients' sample was divided into 3 groups, according to participants' age: group A, composed of 15 subjects aged from 40 to 49 years old; group B, with 14 subjects aged from 50 to 59 years old; group C, composed of 11 subjects aged from 60 to 71 years old.

For aim one, our hypotheses were tested with non parametric analysis of variance (Kruskall-Wallis and Mann-Whitney tests), with age group, gender, global cognitive level, fluidity of navigation and experiences parameters as main independent variables and Virtual Maze results as dependent variables.

Descriptive statistics (mean values and frequencies) were also considered, in order to highlight sample demographic characteristics and Virtual Maze result trends.

With regard to aim two, patients' neuropsychological profiles and the association between cognitive aspects and survey-to-route task performances were qualitatively described, according to a single-case approach.

For aim three, Mann-Whitney analysis was performed in order to compare patients and controls, on the basis of the different age group, with clinical condition (healthy subject vs patient) as independent variable and P&P and Virtual Mazes results as dependent variables. Only two of the age group were considered: 50-59 years old (group B) and 60-71 years old (group C). One patient was excluded from the analysis (patients ID: 5), since she was the only participant belonging to the age group A and she performed well in both tasks.

## Results

### Healthy subjects

All subjects succeeded in completing the 8 P&P mazes.

Mean values and standard deviation (S.D.) of P&P and VR maze results, regarding to the three age groups, are shown in Table 1.

**Figure 3:**
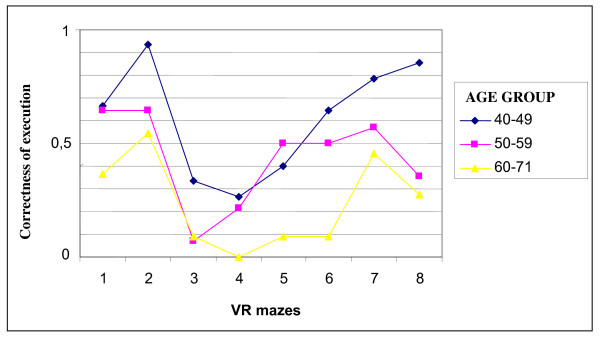
**Age groups performances for single VR mazes**.

As indicated by longer execution times (around 9 minutes for third maze and 8 minutes for fourth maze) and by the number of uncompleted mazes, third and fourth VR mazes resulted to be the most difficult within the eight mazes, with reference to A and B groups. For group C, no subject was able to complete the fourth maze, although a general difficulty was also observed in the third, fifth and sixth maze (see Figure 3).

The effect of age on P&P and VR mazes variables (total execution times and number of mazes completed) was investigated (see the previous paragraph for statistical analysis description).

With regard to P&P mazes, a significant age difference was found for total execution times (p < .03), not for total number of mazes completed (p > .05). Descriptive statistics computed on these data showed a particular trend in group B, who took more time to complete mazes from the third to eight, when compared to both younger (group A) and older (group C) participants. In group B, lower execution times were observed only in first and second mazes execution.

A significant age effect was also found regarding to both VR task variables (p < .05). With reference to the total number of mazes completed, descriptive analysis showed higher scores in group A, compared to group B and C, where older participants obtained the lower scores.

Descriptive statistics, computed on VR execution times, showed a trend similar to that observed with regard to P&P execution times, with higher values in group B, compared to both group A and C.

Nor gender (p > .05) or educational level (p > .05) effect was found for the total number of P&P and VR mazes completed and both total execution times.

On the other side, global cognitive level seemed to have an effect on the total number of virtual mazes completed (p < .05), with a better performance in participants who showed higher Mini Mental State Examination scores. No effect was found for the global cognitive level on P&P and VR task execution times (p > .05).

Besides, total number of VR mazes completed seemed to be influenced by previous experience with computer use (p < .001) and fluidity of navigation into the virtual environment (p < .001). This last variable also had an influence on total P&P mazes execution time (p < .005).

## Patients results

### Neuropsychological results

One patient (ID:5) showed no significant cognitive deficit, except for a mild verbal learning problem, after the recovery from a severe amnesic-confabulatory syndrome, also affecting her topographical orientation abilities.

Another patient (ID:1) showed a verbal fluency deficit and borderline score on the Tower of London test, suggesting the presence of a mild frontal cognitive deficit. Four patients (ID: 2,3,4,6) showed a moderate impairment of attention, memory and executive functions; one patient (ID:7), who showed a visuo-spatial learning deficit and a borderline score on a visuo-spatial planning test, also reported the occurrence of topographical disorientation episodes in everyday life; one patient (ID:8) was impaired in two visuo-spatial tasks, assessing line orientation judgment and visuo-spatial planning abilities (for details about neuropsychological results, see Table 2).

**Table 2:** Patients cognitive and experimental task scores

PATIENTS ID	1	2	3	4	5	6	7	8
**AGE**	65	65	69	71	42	57	57	59

**EDUCATION (YEAR)**	5	5	5	5	13	10	23	5

GENDER *	M	M	F	M	F	F	M	M

LESION **	**LEFT**	**LEFT**	**LEFT**	**BILAT**	**BILAT**	**RIGHT**	**RIGHT**	**RIGHT**

**SITE**	FRONT	FRONT	TEMP	HIPP	THAL	TEMP-PARIET	HIPP THAL	TEMP-PARIET

GLOBAL COGNITIVE LEVEL *** (Raven P.M. 47) Cut off: 18/36	+	+	+	+	+	+	+	E

S-T VERBAL MEMORY *** Cut off: 3.75	+	-	+	+	+	+	+	+

L.T. VERBAL MEMORY *** Cut off: 8	+	-	+/-	+	+/-	-	+	+

S.T SPATIAL MEMORY *** Cut off: 3.5	+	+/-	-	+	+	+/-	+	+

L.T. SPATIAL MEMORY *** Cut off: 6.75	+	-	-	+/-	+	-	-	+

REY'S FIGURE RECALL *** Cut off: 6.3	+	-	+	-	+	+/-	+	+

LANGUAGE COMPREHENSION *** Cut off: 29/36	+	-	+	+	+	-	+	+

PHONEMIC FLUENCY *** Cut off: 17	-	-	+	+	+	+	+	+

SEMANTIC FLUENCY *** Cut off: 25	+	-	+/-	+/-	+	+	+	+

VISUO-SPATIAL PLANNING *** Cut off: 7.75	E	+/-	+/-	E	+	-	+/-	-

PLANNING (TOL) *** Cut off: 16	+/-	E	+/-	-	+	+	+	E

DIVIDED ATTENTION *** Cut off: 186	+	-	+	-	+	+	+	+

SELECTIVE ATTENTION *** Cut off: 33	+	-	-	+	+	+	+	+

GEOGRAPHIC. KNOWLEDGE *** Cut off: 7.5	+	+	+	-	-	+	+	+

RIGHT-LEFT ORIENTATION *** Cut off: 26	25/32	32/32	14/32	23/32	32/32	19/32	31/32	26/32

LINE ORIENTATON JUDJMENT *** Cut off: 15/30	E	-	+/-	+	+	-	+	-

PRAXIA (REY'FIGURE ***COPY) **Cut off: 29**	+/-	+/-	+/-	-	+	+/-	+/-	+

HEMISPATIAL NEGLECT *** Cut off: 129	A	A	A	A	A	A	A	A

**CM_TOT**	8/8	1/8	7/8	8/8	8/8	5/8	8/8	8/8

**CMT_TOT (minutes)**	17,3	32	18,9	20,7	4,21	36,4	10,8	17,9

**VR_TOT**	1/8	0/8	0/8	1/8	5/8	0/8	0/8	0/8

**VRT_TOT (minutes)**	77	60,8	63	74,5	44,3	24,8	56,2	69,7

M: male; F: female.

Front: frontal; temp: temporal; hipp: hippocampal; thal: thalamus; temp-pariet: temporo-parietal.

+: preserved performance (above cut-off); -: impaired performance (under cut-off); **+/-**: borderline performance; E: not administered; A: absent.

### VR Mazes Results

In Table 3, minimum/maximum scores, mean values and standard deviations (S.D.) are presented, with reference to patient performances in P&P and VR mazes. For single patients' results, see Table 2.

**Table 3:** P&P and VR results of Patient group

PATIENTS (TOTAL)	P&PTOT *	P&P T_TOT **	VR_TOT ***	VRT_TOT ****
**Mean**	6,6	19,8	0,9	58,8

**N**	8	8	8	8

**SD**	2,5	10,4	1,72	17,25

**Minimum**	1	4,21	0	24,9

**Maximum**	8	36,42	5	77

*Total number of P&P mazes completed

**Total P&P execution times

***Total number of VR mazes completed

****Total VR execution times

### Comparison between Patients and Control subjects

Mean values and standard deviations (S.D.) of patients' performance, with reference to 50-59 and 60-71 age groups, are reported in Table 4.

**Table 4:** P&P and VR results of Patients, divided into age groups

PATIENTS (AGE GROUPS)		P&P_TOT *	P&PT_TOT **	VR_TOT ***	VRT_TOT ****
	Mean	7	21.7	0	50,26
	
**50-59**	N	3	3	3	3
	
	SD	1,73	13,23	0	23
	
	Minimum	5	10,76	0	24,88
	
	Maximum	8	36,41	0	69,73

	Mean	6	22,2	0,50	68,85
	
**60-71**	N	4	4	4	4
	
	SD	3,36	6,6	0,57	8,13
	
	Minimum	1	17,3	0	60,76
	
	Maximum	8	31,9	1	77,06

*Total number of P&P mazes completed

**Total P&P execution times

***Total number of VR mazes completed

****Total VR execution times

The comparison between 50-59 aged healthy subjects and patients (group B) revealed a significant difference in total VR mazes completed (p < .05) and total P&P mazes completed (p < .05); analysis on single mazes showed that patients differed significantly from the control group with regard to first (p < .05) and second (p < .05) VR mazes and third, fourth and eighth P&P mazes. Significant differences were also found with regard to P&P execution times; single mazes analysis showed that an interaction effect was present only for the first maze.

The comparison between healthy subjects and patients samples aged 60 to 71 years old showed a significant difference in total number of P&P mazes completed, with particular reference to the third maze (p < .05) and to total P&P mazes execution times, for all mazes except for the sixth (p < .05).

## Discussion

The present study has investigated how age related cognitive decline affects performances in a task requiring the transfer from survey to route representations in a virtual environment. Besides, it has evaluated how cognitive difficulties consequent to brain lesions can affect this ability into the same virtual environment.

With regard to healthy subjects, our results showed that a clear effect of age was evident in survey to route transfer and that this ability depends, at least in part, on global cognitive level, whereas it is not influenced by the level of education and by gender. As predictable, we also found an influence of previous experience with the use of computer and easiness to use (that we named "fluidity of navigation") in VR test performance.

Moffat and Resnick (2007) argued that successful navigation relies on pre-frontal dependent executive skills, such as behavioural monitoring and cognitive flexibility. These abilities are shown to decrease with aging, together with working memory ability and speed of processing. Our results can be interpreted according to Moffat and Resnick hypothesis, that emphasizes the role of non-spatial memory components of behaviour in age differences observed in place learning and spatial navigation tasks.

Consistent with this hypothesis, we found a clear correlation between global cognitive functioning and healthy subject performances with regard to the ability to transfer survey information into egocentric routes. This could explain the significant variability observed in elderly population with respect to topographical abilities: the maintenance of a high cognitive functioning seems to interact with aging process, probably offering compensatory strategies to age-related decrease of executive processes efficacy.

Since the eight mazes differ with respect to structure complexity, some considerations can be drawn from the observation of single mazes executions. In particular, participants older than 61 years, compared to younger subjects, showed more difficulty in performing fifth and sixth VR mazes.

The sixth VR maze requires a 180° turn around oneself at the starting point in order to take the correct direction toward the exit point. A greater cognitive load, in particular spatial rotation skills, is required in order to do not get lost at the first steps. Spatial rotation ability has been shown to represent an age-dependent function, according to working memory impairments observed in elderly samples.

We argue that age differences observed in this study were not due to the lack of computer experience and generalized psychomotor slowing in the older participants, for two reasons. First, all participants were given training about the use of the keyboard for navigating into the VR mazes, and subjects who were not able to learn basic movements into the virtual environment were excluded. Second, even if experience with computer and fluidity of navigation were observed to have an effect on task execution, these variables were similarly distributed among the different age groups, at least for group B (50-59 years old) and C (60-71 years old).

According to previous studies, visuo-motor and experience-based components of virtual navigation primarily affect time measures. In our study, these variables seemed to be significantly related to total number of mazes completed, not to execution times. On the other side, execution times do not represent an effective parameter in the VR task, since they do not allow to discriminate between age groups or between healthy subjects and patients' performances. We argue that longer execution times observed in the middle aged group (50-59 years old), compared to older participants, may be related to a stronger effort, in order to reach the maze exit and successfully complete the task; on the other side, many older participants refused to continue with the maze as soon as they got lost, feeling soon discouraged. In support to our hypothesis, this tendency was observed, in particular, in the third maze, which represents the most difficult trial, requiring a change of strategy. A similar performance was observed for P&P task, where middle aged participants took more time, with respect to the older ones, to complete the 8 mazes. In this case, this result may also suggest the presence of a ceiling effect for the P&P task. Since we introduced this task as an intermediary step for VR mazes execution, these results cannot be considered a limitation of our study.

With regard to patients' group, also considering the small number of participants, a view of their performances at experimental task and neuropsychological results seems to help in drawing some preliminary considerations.

Impairments in VR task execution were observed in 7/8 patients, when compared with age-matched healthy subjects. The only subject who showed a preserved performance, completing 5/8 VR mazes, was a patient (ID:5) without significant cognitive deficit, except for a mild verbal learning problem, after a recovery from a severe amnesic-confabulatory syndrome also affecting her topographical orientation abilities.

Regarding to the other subjects, two main neuropsychological profiles could be identified: two patients (ID: 7 and 8) showed visuo-spatial functions deficits, whereas the other patients suffered from a more global impairment in attention and executive functions (ID: 1-4 and 6).

No clear differences were observed with reference to lesion sites.

From these preliminary results, we argue that both a selective deficit related to visuo-spatial skills and a more global compromised neuropsychological profile affecting strategic reasoning and cognitive flexibility, may contribute to influence the performance in the complex survey to route task.

## Conclusions

The VR maze task efficiently highlighted the role played by aging in the transfer from survey to route topographical representations. VR task was also useful in order to discriminate between healthy and brain damaged subjects with regard to 50-59 years old participants, whereas no significant differences were observed when considering the oldest subjects (even if patients' group performed worse than matched healthy subjects in this age range).

Our study provides empirical support to previous theories which argued that aging process affects the effective management of complex orientation tasks.

Otherwise, it is necessary to underline some limitations: first, more patients would be useful in order to draw quantitative considerations about cognitive and survey-to-route task performance associations. Second, the inclusion of 40 to 71 aged healthy participants has left out a significant proportion of the general population, with critical implications for results generalization. Finally, the administration of the neuropsychological battery even in healthy subjects may provide additional data in order to make correlation between traditional cognitive and VR measures and to identify the most suitable cognitive tests to underline problems with topographical abilities.

Besides, the task involved in our study doesn't simply require the transfer from survey to route perspective; it also involves computer use, with which older persons are not usually accustomed. The lack of self-confidence with experimental task could have influenced real abilities, worsening performances. For this reason, we introduced two additional variables (experience with computer and fluidity of navigation into the virtual environment) in order to examine this important aspect.

Human-computer interaction issues and subjective experience should be always considered when new technologies are used with elderly population and neurological patients, basing on the assumption that the individual, society and technology are interacting dimensions in constant evolution.

## Competing interests

The authors declare that they have no competing interests.

## Authors' contributions

LC and MLR conceived the study rationale and design, participated to the study coordination, performed the statistical analysis and contributed to draft the manuscript. FM and CSt contributed to the sample recruitment and preliminary data collection. CSc participated in the experimental test administration. GR represents the head of the Applied Technology for Neuropsychology Laboratory, providing the knowledge and instruments necessary to the conceptual and technical VR task development.

All authors read and approved the final manuscript.
